# Attitudes and Beliefs Towards Ration Planning Among German Organic Pig and Poultry Farmers

**DOI:** 10.3390/ani15060807

**Published:** 2025-03-12

**Authors:** Margret Krieger, Susanne Hoischen-Taubner, Leonie Blume, Albert Sundrum

**Affiliations:** Department of Animal Nutrition and Animal Health, University of Kassel, Nordbahnhofstraße 1a, 37213 Witzenhausen, Germany

**Keywords:** feeding, nutrition, organic agriculture, pig, poultry, knowledge

## Abstract

Providing young monogastric animals with the essential amino acids they need is challenging in organic feeding. The nutrient content of organic feeds varies widely, and additives are restricted, so feed rations must be formulated based on analysis. A survey of 56 organic pig and poultry farmers in Germany was carried out to investigate their attitudes towards ration planning and to examine and evaluate the feeding management on their farms. Principal component analysis was used to characterise the different attitudes. Cluster analysis revealed three groups: Farmers in Group 1 (n = 28) were uncertain about the nutrient requirements of organic animals. They accepted the importance of need-based feeding for health and performance, but did not recognise the importance of regular ration adjustments and were uncertain about the animals’ ability to compensate for deficiencies. In Group 2 (n = 11), need-based feeding and regular ration adjustments were most important to farmers, who were more likely to have professional feeding systems. In Group 3 (n = 17), farmers were least uncertain about nutrient requirements and did not believe that animals would lower their demand when undersupplied. Although the importance of need-based feeding was recognised, regular ration adjustment was of little importance for this group. Across all farms, 60% of the feed rations examined deviated significantly from recommendations. It is concluded that organic farmers need more guidance and perhaps pressure to fulfil their responsibility to provide their animals with the nutrients they need.

## Simple Summary

In organic farming, it is often a challenge to provide young pigs and poultry with the nutrients they need because of limited feed sources and restrictions on the additives that can be used. Asking pig and poultry farmers in Germany about their attitudes and beliefs towards ration planning and examining their feeding management, we found that groups of farmers were characterised by similar attitudes. We found that many farmers were uncertain about their animals’ needs and the consequences of inappropriate feeding. In line with this, we found that many feed rations deviated significantly from feeding recommendations. We conclude that many organic farmers would benefit from more guidance on feeding for the benefit of the animals in their care.

## 1. Introduction

It is well known that farm animals have specific requirements for nutrients. The dietary needs of an animal depend on several factors, i.e., the requirement for maintenance of body functions and the requirements for performing specific tasks, such as growing, reproducing, suckling, mating, keeping warm, moving about, fending off pathogens, etc. Depending on the species, life stage, performance level, individual condition, and environment, an animal’s nutritional requirements can vary widely [1,2]. Feeding farm animals according to their dietary needs serves several purposes; it enables optimal development and performance, promotes animal health and normal behaviour, and allows for efficient use of resources, thus helping to preserve the environment.

Young animals have a particular need for appropriate feed composition. They grow rapidly, resulting in a high requirement for essential nutrients, while at the same time, their feed intake capacity is limited by their small body size [3]. Most demanding is the feeding of young monogastric animals. While young ruminants, once they enter the ruminant phase, can draw upon their symbiosis with rumen microbes, young monogastric animals entirely depend on the intake of an adequate quantity and proportion of essential amino acids with their feed. This poses a challenge, especially in organic farming, where synthetic amino acids are not permitted, conventional protein sources (e.g., side products with favourable amino acid patterns, such as potato protein) are restricted, and organic purchases are limited by Regulation (EU) 2018/848 [4]. High-quality protein sources of organic origin, such as grain legumes, oil cakes, and other side products, are generally available, but their quantity is limited, and they come at high costs [5]. In addition, conflicts with environmental objectives arise when organic components, such as soya, are imported from overseas [6].

The formulation of feed rations is closely linked to the animal health status [7]. In monogastric animals, an undersupply of essential amino acids, due to their importance for protein synthesis, results in reduced development and performance. It can also lead to abnormal behaviour, such as increased feather pecking, tail biting, and cannibalism, as well as developmental disorders and health issues [8,9]. High proportions of grain legumes with relatively low essential amino acid content can lead to an oversupply of nonessential amino acids and higher excretion of nitrogen. As a result, feed conversion decreases, and the metabolism is excessively stressed [10].

Due to highly heterogeneous nutrient contents and unfavourable amino acid patterns in regional organic protein components such as cereals, pulses, and oil cakes, the formulation of appropriate feed rations based on feed analyses, combining numerous ingredients to achieve the desired nutrient levels, is therefore of major importance [11,12]. Animal nutritionists have been teaching this for decades [13,14], yet the level of implementation by organic livestock farmers is still questionable.

The aim of our study was to add to the understanding of the drivers and barriers to the uptake of need-based feeding strategies, investigating German organic pig and poultry farmers’ attitudes and beliefs towards ration planning. Such an understanding may inform the debate on prospective approaches to improve need-based feeding in organic farming and, as a consequence, may help to improve health and welfare in organic livestock systems.

## 2. Materials and Methods

### 2.1. Acquisition of Farms

The acquisition of the farms involved in the study was carried out by organic consultants. In order to identify potentially interested farms, the project was introduced to farmers, advisors, and a professional audience on various occasions, e.g., livestock conferences. Furthermore, articles were placed in farmer magazines and newsletters, and individual persons were addressed directly. The study farms were selected with the aim of including different animal species and feeding strategies in the study and must thus be regarded as a purposive sample.

### 2.2. Farm Characteristics and Feeding Strategies

Organic consultants carried out the farm visits for data collection in the period between May and September 2017. Before the visits, the assessment protocol was tested, together with the consultants, in a pretest on a farm that did not belong to the group of project farms. During the actual visits, farmers were interviewed about the performance and husbandry conditions of their livestock, their feeding strategies, and the crop production potential of the farm. In addition, key figures on the costs and revenues of livestock production were requested. Data was collected through a structured protocol that could be completed using a paper version or by an online survey administered through LimeSurvey [15].

During the farm visits, in addition to the inspection of the local conditions, samples of all feeds used on the farm (individual components, complete feeds, supplemental feeds, and self-mixed rations) were taken for the purpose of assessing the current supply situation of animal husbandry. Wet chemical analyses of crude nutrients and energy were performed for single components according to the methods of VDLUFA [16]. Where no sample could be obtained, table values for crude nutrients were used. For complete and supplementary feeds, declarations were used to derive crude nutrients.

### 2.3. Need-Based Feeding

With regard to the performance of the animals, the extent to which the feed rations were suitable for meeting the needs in their respective development phases was examined. As animals of conventional genetic origin were predominantly kept on the farms, the German recommendations for nutrient supply for different performance levels were taken as a reference for the evaluation of feed rations and ration planning [17,18,19,20]. Deviating from this, lower energy levels were used for the evaluation of the need-based feeding of laying hens, which is a common practice amongst organic farmers to increase the amount of feed consumed. For broilers and turkeys, results from feeding trials under organic feeding regimes were used [21,22,23].

Reference values for energy and crude protein supply requirements for the respective animal groups were thus determined based on literature and expert knowledge (Table 1). The evaluation of the needs coverage of feed rations, i.e., the nutritional mismatch between supply and demand, was carried out by comparing the feed offered at the respective development phase with recommended values and classifying them into one of three categories: (i) within the tolerance range (10% above and below the recommendation), (ii) undersupplied (deviation of more than 10% below the recommendation), or (iii) oversupplied (deviation of more than 10% above the recommendation).

### 2.4. Attitudes and Beliefs Towards Need-Based Feeding and Ration Planning

Attitudes and beliefs towards need-based feeding and ration planning were assessed outside the farm visits through a separate survey. This was to reduce response bias due to desired response behaviour. The survey could be taken using a paper version or by answering an online questionnaire also administered through LimeSurvey [15]. It comprised 35 mainly closed questions, one of which asked respondents to react to 17 statements on need-based feeding. The statements were designed based on answers and points of view given in the first round of interviews by farmers and on the feedback from the advisors participating in the project.

Respondents could indicate their agreement with the statements on a five-point Likert scale, from left to right, ‘Strongly agree’, ‘Agree’, ‘Neither agree nor disagree’, ‘Disagree’, and ‘Strongly disagree’. For analysis, the indications of agreement were translated into scores (ranging from +2 for ‘Strongly agree’ to –2 for ‘Strongly disagree’). Scores of items 2 (‘*Animals are able to compensate even insufficient supply*’), 3 (‘*Good performance is possible even with a feed supply below the animals’ needs*’), 5 (‘*Savings in feed purchases maintain the economic advantage, even when animals cannot deliver their maximum performance*’), and 16 (‘*Nutrient deficiencies do not harm the animals because they adapt their performance level to the feed offered*’) were then reversed. This was performed to align them with the other variables (where stronger agreement puts more emphasis on a need-based diet).

### 2.5. Principal Component Analysis

Principal component analysis (PCA) was used to reduce the number of original variables (items) to an independent set of variables characterising attitudes toward the importance of need-based feeding. PCA was performed using the psych package in R [26]. Kaiser–Meyer–Olkin criterion (KMO) was used to test whether sufficiently large relationships exist within the data set to perform PCA. KMO was 0.741, and the diagonals of the anti-image correlation matrix were greater than 0.5, indicating that the data set is suited [27,28]. Bartlett’s test of sphericity showed that the variables were not completely uncorrelated (χ^2^ (136) = 490.07; *p* < 0.001), allowing for PCA to be carried out.

PCA identified four components that together explained 71.4% of the variance (see Table 2). Both the Kaiser criterion and visual scree plot analysis were used to arrive at a number of components that accurately and understandably explain the observed correlation matrix. PCA results were rotated using an oblique rotation method (Oblimin).

Initially, 17 assessed items were used for PCA. In the course of the analysis, two of these items were removed. Item 1 (*‘Optimal performance is only possible with feeding according to animals’ needs’*) had a component loading of 0.57 on component 4 (which was well defined by three other items) and a cross-loading of 0.27 on component 3. Item 12 (‘*Table values for the nutrient content of feed are a sufficiently accurate basis for ration calculations’*) had component loadings of 0.41 and 0.56 on component 1 and 2, respectively. Item removal was undertaken in accordance with the 0.40–0.30–0.20 rule [27], except for items 13 and 16, where some cross-loading was tolerated (the primary loading was relatively high and cross-loading was relatively low, leading to a difference of >0.20).

Final primary component loadings were >0.58 for all items. As shown in Table 3, for most items, communality (the fraction of the variance that is accounted for by the components) was higher than uniqueness (the remaining variability). According to Hoffman’s index of complexity [29], an item specific to a component should have an item complexity close to one, whereas high item complexity indicates cross-loading. The mean item complexity was 1.2. As explained above, some cross-loading was tolerated based on conceptual considerations.

For each of the components, composite values were formed by averaging the items that had their primary loadings on the respective component. These were later used for cluster analysis. The composite score data in the current study had an approximately normal distribution, making the data well suited for parametric statistical analyses.

### 2.6. Hierarchical Cluster Analysis

Cluster analysis was used to (i) identify groups of farmers with matching attitudes and (ii) describe those groups in terms of their similarities (and differences). In order to identify robust clusters, based on the components identified by the PCA, a hierarchical cluster analysis (based on the Ward D2 method) was carried out using the stats package in R version 4.4.2 [30,31]. The optimal number of clusters (k = 3) was estimated by evaluating the dendrogram and by applying the methods “wss” (for total within sum of square) and “gap_stat” (for gap statistics) as part of the factoextra package [32]. Standardisation of variables was not necessary because components had identical scales.

Once clusters were determined, the relationships between components (of attitudes) and clusters (of farmers) as well as the characteristics of clusters were analysed using the “describeBy” function of the psych package [26]. Differences between clusters were statistically analysed by one-way ANOVA with heteroscedasticity consistent (HC) standard errors, pairwise *t*-test, and Tukey’s HSD (Tukey Honest Significant Differences) test using the lmtest package [33] and the sandwich package [34,35]. For variables that were not normally distributed, the Kruskal–Wallis test was used, followed by post-hoc tests (pairwise Wilcoxon test and Dunn-Bonferroni test). Where nominal variables were concerned, differences were statistically analysed by the χ^2^-test. This was performed using the base and stats package [31] as well as the rstatix package [36]. All tests were performed at a confidence level of 95%.

### 2.7. Description of Sample

Data used in this study came from 36 organic pig and 20 organic poultry farms in 12 different counties in Germany. The demographics of the sample of farms are shown in Table 4. It reveals that, as planned, farms with different herd sizes, feed bases, and feeding strategies were examined.

Within the group of project farms, not only herd size and management parameters but also performance levels of the animals varied substantially (see Table 5). For turkey farms, the evaluation of performance was difficult due to data gaps.

More details with regard to the feeding management and the economics of the studied farms were described and discussed thoroughly by Blume et al. [12].

## 3. Results

### 3.1. Importance of the Farm Branch

From an economic perspective, the farm branch considered in the project (pig, egg and/or poultry production) was of great or very great importance for two-thirds of the farm managers. This assessment was particularly true for piglet producers (88% of farms), pig fattening farms (68%), and turkey farms (66%). For five of the nine egg producers and three of the five broiler farms, the branch of operation was only of minor or very minor importance. Only 29% of all farms employed labour that specialised in livestock production.

### 3.2. Need-Based Feeding

A total of 139 individual components and 147 mixtures, i.e., complete feeds (43), supplemental feeds (28), and self-mixed rations (76), were analysed with regard to nutrient content. Samples within the same single feed components differed, sometimes considerably, in both crude protein and energy content (see Table 6). The mean values were in the order of magnitude to be expected in organic farming [37]. If the minimum and maximum contents are considered, there were differences of 4–11% within an individual component depending on the farm and location.

As was shown in Table 4, the studied farms pursued different feeding strategies, i.e., self-mixing and the purchase of complete feeds. A combination of the two strategies was also found. In most of these cases, complete feed was purchased for young animals (piglets and chicks), and feed rations for older animals were mixed by the farmers themselves.

Most of the project farms stated that they had their feed analysed for nutrient content at least occasionally (80.4%). In contrast, just under 20% relied exclusively on table values. The largest proportion of project farms had some feed analysed at irregular intervals as required. Only 14.3% of the project participants had their own feed analysed every year and 5.4% also included purchased feed. In the case of purchased feed, the majority of the project participants (78.6%) trusted the declaration and did not have any verifying analyses carried out. Only in the case of farms with laying hens did more than half of the farms state that they had at least randomly commissioned the testing of purchased feed.

Calculations of feed rations were mostly carried out if there were problems in the herd (42%) or if the composition of the feed components changed (31%). A total of 7 out of 56 farms (including four farms that used complete feed) did not carry out any ration calculations. Only 15% of the farms stated that they regularly recalculated feed rations once or several times a year. With the exception of one laying hen farm, these were exclusively pig farms. The most common reasons given for changing the feed ratio were that cheaper components were available (48%) or that they were responding to a recommendation from advisors (42%).

In pig fattening and sow husbandry, at least two-phase feeding was implemented on the majority of farms. However, only four out of the 17 piglet producers fed the recommended three phases (see Table 7). Two farms did not offer any piglet feed. Only one out of nine egg producers adapted the hen ration to changing requirements during the laying period. In broiler farms, universal fattening until slaughter was used in the majority of cases after the starter phase. In turkey fattening, two of the six farms implemented the recommended four-phase feeding.

As shown in Table 8, the feed rations showed a wide range of nutrient contents across all animal species. In fattening pigs, it became apparent that not all animals were adequately supplied with crude protein in the prefattening rations. In the final fattening phase, on the other hand, the potential to significantly reduce the crude protein and energy content often remained unused. After chick age, broilers were mostly fed a universal fattening diet. Energy contents of just above 9.0 MJ ME, which were found on three farms, were classified as very low and did not cover requirements. The turkey rations showed balanced energy contents and very heterogeneous crude protein contents. The feed rations for laying hens revealed the least variation in crude protein content. While pregnant sows were on average over-supplied with both crude protein and energy, lactating sows were often under-supplied in terms of covering their crude protein requirements.

Of 163 feed rations considered for pigs and poultry at different stages of development, 98 feed rations (60%) deviated by more than 10% from the recommended levels of energy and crude protein for the respective age group. Deviations in protein content (65 feed rations) were more frequent than deviations in energy content (33 feed rations). In 19 feed rations (11.7%), the energy content was undercut by more than 10%. In 14 feed rations (8.6%), energy content was more than 10% above the supply recommendations. Crude protein content fell short of the supply recommendations by more than 10% in 41 feed rations (25%). This mainly concerned feed rations for animals with a high protein requirement (young stock, lactating sows, and laying hens in phase I). Twenty-four feed rations (14.7%) contained more crude protein than the animals needed for the corresponding development phase. This was the case, for example, with 10 feed rations for pregnant sows and 10 feed rations for finishing pigs.

### 3.3. Attitudes Toward Need-Based Feeding

**Figure 1:**
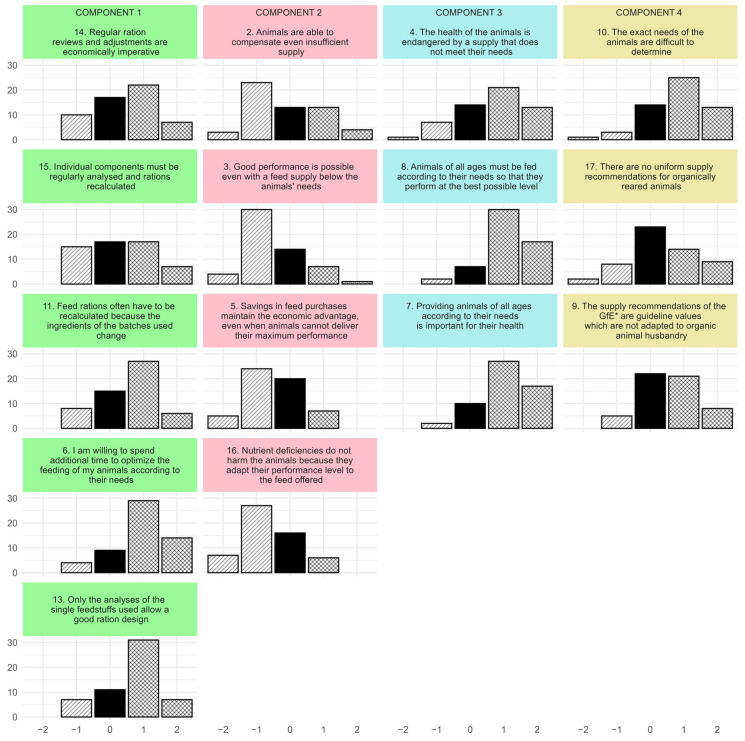
Farmers’ responses (counts) to statements about need-based feeding ranging from strongly disagree (−2) to strongly agree (2), bar pattern indicates disagreement/neutrality/agreement, items are sorted by components (vertical blocks) and by factor loading (highest on top) (n = 56), * GfE = German Society of Nutrition Physiology.

Farmers’ responses to statements about need-based feeding are shown in Figure 1. The majority of respondents were convinced that providing animals of all ages with the nutrients they need is essential for their health and performance. The respective statements 4, 7, and 8 were assigned to component 3 (‘health and performance’). It stands for an attitude that assumes that inappropriate feeding can have undesired consequences for both the health and the performance of the animals, and that need-based feeding is a prerequisite for both production goals.

Most of the respondents were also of the opinion that different nutrient levels and feed compositions require regular analyses and ration adjustments and were willing to spend more time optimising their feeding. The associated statements 6, 11, 13, 14, and 15 were assigned to component 1 (‘regular adjustment’). In summary, this component describes an attitude that attaches great importance to regular adjustments of feed rations based on analyses of the components used. Variables with high loading on this component express that due to the fluctuating ingredients of components, the time spent on ration control and optimisation is an economic necessity.

While the majority of farmers disagreed, a small number believed that animals were able to compensate for inadequate nutrition (e.g., due to using home-grown components with unfavourable nutrient levels) by reducing performance and that reduced performance was economically outweighed by reduced feed costs (statements 2, 3, 5, and 16). Component 2 (‘compensation’), therefore, resembles an attitude that is based on the conviction that animals can compensate for an undersupply, e.g., by adjusting their performance, and are therefore not adversely affected by a shortage of nutrients. Items with a high loading on this component also express that an undersupplied animal can still perform sufficiently and that a potential underperformance would be compensated for by lower feed costs.

There was much uncertainty regarding the supply recommendations for animals in organic husbandry and the suitability of the recommendations of the German Society for Nutritional Physiology (GfE) for organically reared animals (statements 9, 10, and 17). Component 4 (‘uncertainty’) describes an attitude characterised by uncertainty about the needs of animals under organic farming conditions. It summarises items that recognise the lack of organic supply recommendations or the inapplicability of existing recommendations and the resulting problems in determining needs.

Despite the general agreement and disagreement described before, there was also a significant proportion of farmers who neither agreed nor disagreed with the statements presented to them. This hints at a general uncertainty in terms of need-based feeding within the surveyed group of farmers. Furthermore, it must be noted that even when a majority agreed or disagreed with a given statement, there always existed at least some farmers with an adverse opinion.

### 3.4. Groups of Farmers with Similar Attitudes

Hierarchical cluster analysis resulted in a three-cluster solution with highly significant differences between clusters regarding the expression of components (see Table 9).

The first cluster of farmers (CL1) of the three groups was quite unsure (or divided) about the animals’ capacity to compensate for nutrient deficiencies by reducing their performance and thus coping with undersupply. This is shown by the group’s mean composite score, which lies very much in the middle of total acceptance (–2) and total rejection (+2) of the idea (values were reversed for all items loading on component 2, see Materials and Methods). The group was also the most uncertain of all groups in terms of organic animals’ nutrient requirements. Regular feed analyses and ration adjustments were only deemed to be of slight importance, whereas the group somewhat accepted the importance of need-based feeding as a basis for health and performance.

Regular ration adjustments were considered very important by the second group of farmers (CL2) and the idea of animals reducing their requirements was strongly rejected. There was a strong belief that nutritional adequacy was a prerequisite for animal health and performance and some uncertainty about the nutritional needs of organic animals.

The third group (CL3) was least uncertain about the feeding requirements of organic animals. Regular analyses and ration adjustments were considered to be of little importance. The concept of compensation was rejected, but not as strongly as by CL2. The importance of need-based feeding for health and performance was only weakly recognised by this group.

As can be seen in Table 10, the different animal species and purposes were represented in almost all clusters. Pig farmers were mainly found in CL1 and CL3, with more fattening farms in CL3 and more piglet producers in CL1. The majority of egg and broiler producers were found in CL1, while turkey farmers were evenly distributed between CL1 and CL2. The three part-time farms (two broiler farms and one pig fattening farm) were also evenly spread across all clusters.

Cluster-describing variables are shown in Table 11. There was no difference between clusters in terms of agricultural area (an indicator of farm size) and the duration of organic farming (time since conversion). Farms having divided responsibilities between crop and animal production were evenly distributed (no statistical difference). The farm branch under study was equally important to the surveyed farmers.

When management practices were reported by the farmers, some differences between clusters became apparent. The verification of feed declarations did not differ. However, most CL1 farmers had feed analyses performed sporadically (2 = *‘I only have some feed analysed at irregular intervals if necessary.’*) while CL3 farmers had them performed on a regular basis (3 = *‘I have several feedstuffs analysed each year.’*) but were still far from having all feed components analysed on an annual basis. CL2 farmers lay in between. A comparable pattern appeared regarding the frequency of ration calculations, where CL1 scored comparably low (2 = *‘Irregular, only in case of problems in the herd.’*) and CL2 and CL3 scored, on average, somewhat higher (3 = *‘Only if the composition of the feed components changes.’*), but still only just above medium related to the scale. Ileal amino acid digestibility was poorly considered by CL1 farmers (1 = *‘No, I do not carry out an evaluation’*) and only partially considered by CL2 and CL3 farmers (2 = *‘Yes, based on estimated values for some feed components.’*, 3 = *‘Yes, based on estimated values for each individual feed component.’*), but was never analysed. Information on phase feeding was collected for fattening pigs and laying hens. All but one of the laying farms fed one phase during the laying period. The one farm belonged to CL1. Due to the small sample size, no statistical test could be performed with this variable. Most pig-fattening farms in CL3 fed two phases, while most farms in CL2 fed three or four phases. However, the apparent differences were not statistically significant.

Performance measures for piglet production, pig fattening, and egg production indicated higher performance levels in CL2 and CL3, but statistical tests did not confirm this (probably due to the small sample sizes). Similarly, problems such as feather pecking, tail biting and cannibalism (combined), and diarrhoea, as well as low performance were reported predominantly by CL1 farms, but tests did not reveal statistical differences between clusters. Feather pecking, tail biting, and cannibalism were not reported for laying hens and pigs in closed systems but for all other animal groups (sows, fattening pigs, broilers, and turkeys). Diarrhoea was mostly reported for poultry and not at all for fattening pigs and pigs in closed systems. Low performance was reported by four farms (three egg producers and one piglet producer).

## 4. Discussion

Analyses of feed rations in organic pig and poultry farms showed that a considerable share deviated more than 10% from recommended nutrient levels. Similar findings have been reported by Thielen and Kienzle [38]. More recent field studies are rare, and yet they are of great value. The identified mismatch between feeding practice and recommendations is worrying and can have many different reasons [39]. One could be that organic farmers are not guided by the respective recommendations, but pursue their own (self-referential) strategies. The other could be that farmers do want to follow recommendations but fail to create the targeted feed rations for various reasons.

### 4.1. Recommendations, Knowledge, and Beliefs

The farmers’ responses to the statements showed that (i) farmers were uncertain about the exact needs of their animals, (ii) a large number of farmers did not consider the official GfE recommendations to be appropriate for organic animals, and (iii) many farmers were unaware of existing recommendations on how to meet nutritional needs with organic feed ingredients. It is true that most nutritional requirements refer to the performance level of modern genetic origins under conventional housing and feeding conditions [18,19,20,40,41]. Such recommendations, in order to be applicable in organic systems, must be translated to account for the actual performance level of the animals kept and the additional demands for temperature regulation in outdoor climate systems, movement and foraging in free-range systems, etc. Such ‘translation’ can, however, quite easily be implemented as factorial calculations exist. It requires a minimum of information on the housing conditions and of the animals themselves. The latter can be a problem in non-specialised organic farms, where especially animal data (live weights, performance data, etc.) are often lacking. Within the project, performance levels showed a wide range. Depending on the animal species and age class, considerable deviations from the level of previous publications on performance levels in organic livestock production were identified [23,24,42,43,44]. Once requirements have been established, tools are available to help formulate the appropriate diet, but there may be a need for more low-threshold solutions. There are also a number of practical guides to organic feeding. However, our results show that they have not been sufficiently incorporated into the feeding practice of the majority of organic pig and poultry farms.

The majority of farmers appear to lack the data, knowledge and skills, and/or the resources needed to determine the nutritional requirements of their animals and to provide the required nutrients through a targeted combination of available feed ingredients. As a result, many feed rations in the study farms deviated significantly from the recommendations. Our analyses have shown that nutrient levels varied widely among individual components. A successful strategy, however, depends on knowledge of the ingredients of the feed components used. A comprehensive chemical analysis of the protein carriers relevant for feeding pigs and poultry in organic farming, including in vitro digestibility, has been carried out by Kyntäjä et al. [45]. However, the table values resulting from the analyses can only provide orientation and guidance. Due to a high variation of ingredients between feed batches, the use of table values is not immune from leading to wrong conclusions [46]. Therefore, the analysis of at least the farm’s own feed batches is considered essential [47]. Farmers’ responses to the given statements show that many of them are aware of varying nutrient levels and the need for regular analyses and ration adjustments. Yet, there seems to be an intention-behaviour gap, where resource limitations (time, money, tools, etc.) hinder implementation. Farmers may be unaware, that inefficient feeding can cost them much more than the cost of some feed analysis.

The degree to which animals were supplied according to their needs was closely related to the design of feeding phases in the studied farms. Feeding phases allow the nutrient content to be adapted to the average needs of the animals in a feeding group [48]. Many of the project farms did not make use of this possibility of adjustment. Lack of knowledge and/or resource limitations in connection with small animal numbers may be reasons why phase feeding is not implemented as recommended.

Responses to the given statements showed that many organic farmers believed that need-based feeding is crucial for the health and performance of monogastric animals and that a wrong supply puts both at risk. Some farmers were, however, convinced (or at least uncertain) of the idea that animals could, to a certain extent, tolerate an inadequate supply and could even downregulate their performance to prevent them from harm. Due to differences between the composition of organic and conventional feedstuffs and feeding regimes, there is evidence that animals of the same genetic origin can perform differently [49]. It has also been shown that animals can reach their target weight despite being fed slightly different levels of nutrients [44,50]. However, the capacity to downregulate their demand may be partly overestimated by farmers and somewhat serve as an ‘excuse’ for not providing sufficient nutrients, while still compromising animal health, e.g., in weaners and sows [38], and contributing to behavioural abnormalities such as feather pecking, tail biting, and cannibalism [51,52]. Although it has been discussed for some time and the term ‘robustness’ is widely used (with regard to organic livestock), there remains a knowledge gap concerning the degree to which animals can adapt to a variation in nutrient intake, i.e., the limits of feeding for welfare [10]. On the other hand, diet-related illness serves as a discernible indicator of an overstressed capacity to cope with nutrient imbalances [1,53]. Accordingly, the farm-specific prevalence of diet-related diseases can and should be used as a metric to evaluate the adaptability of animals in the farm-specific context (compared to average figures for organic farms), and to provide guidance for the farm management on the necessity of interventions.

### 4.2. Feeding Management to Meet Animals’ Needs

The limitation of nutrient availability to on-farm and organically produced feeds poses major challenges to many organic farms in their efforts to meet the nutrient requirements of high-yielding pigs and poultry. This is especially true for the supply of essential amino acids to young monogastric animals [54]. In a report on organic protein availability in Europe, the degree of self-sufficiency for crude protein is estimated at 56% on average for 10 European countries (Austria, Denmark, Finland, France, Germany, Lithuania, Netherlands, Sweden, Switzerland, United Kingdom) [55]. In contrast, the authors estimate the degree of self-sufficiency in Germany to be approximately 64%. However, the degree of self-sufficiency with regard to the essential amino acid methionine is classified as particularly low at approx. 47%. Based on various assumptions, the authors roughly calculated a total methionine requirement for organically raised pigs and poultry of approx. 812 metric tons per year. The gap in high-value protein sources, especially those containing methionine, is correspondingly high.

A lot of research has been conducted to identify and test alternative feed sources of high-quality protein, such as insects, seaweed, and legumes [5,56,57,58,59]. Cereals, and in particular wheat and wheat by-products, can make a relevant contribution to protein supply. Recent studies show a high digestibility of essential amino acids for pigs depending on the variety [60]. However, due to the variation between varieties, there is a need for analysis of individual components and targeted supplementation of deficient amino acids. The same holds true for the wide selection of other components that are generally available to organic farmers. Without the possibility to incorporate synthetic amino acids, nutritional requirements of high-yielding hybrid genetics can only be met if the actual ingredients and their digestibility are known and components are carefully combined.

Feed costs in organic pig and poultry farming account for approximately 60–70% of total production costs [45]. Therefore, calculating the costs in relation to the benefits that can be achieved in the respective farm context is another important element for optimizing the management of nutrients [44]. In addition to purchasing protein sources, farmers have various management options (including multiphase feeding) to adapt production processes to the limited availability of essential amino acids [61]. These options have to be brought into line with the farm-specific, in practice often very heterogeneous initial conditions on organic farms [62].

Need-based organic pig feeding can usually be accomplished by using grain and green legumes as well as oilseeds in combination with an adapted phase feeding [42,61,63,64,65,66]. Special attention should be paid to the supply of piglets and lactating sows, as these require feed rations with high-quality feed components with high digestibility. Several publications recommend piglet rearing in at least three phases [20,24]. This appears to be particularly induced in sows with high piglet numbers. Among other things, it increases feed intake in the piglets and protects the sows from excessive decomposition of body substance, which remains a risk factor in organic sows [62].

Due to the organic framework conditions, an adequate supply of essential amino acids is a challenge. Often, these are not present in the recommended concentration in the feed ration. This is especially true in poultry feeding. Suppose poultry is fed under organic feeding conditions at the energy levels recommended for conventionally raised poultry. In that case, feed intake is often insufficient to ensure a need-based supply of essential amino acids, particularly methionine. This can have far-reaching consequences, such as declines in performance, behavioural abnormalities such as feather pecking and cannibalism, lowered animal health levels, and loss of income [52,67]. Poultry may be stimulated to increase feed intake via a reduced energy content of the diet, thereby increasing the supply of essential amino acids [21,22,23,59,68].

Various studies have shown that 100% organic feeding with different protein sources is possible [21,44,61,69], but requires additional and often costly efforts. The challenge here is the constant availability of different feed components, the handling of heterogeneous qualities and their specific usage within the farm. In order to master the various challenges of need-based feeding under organic conditions, the availability and evaluation of farm data play a key role [70].

### 4.3. Groups of Farmers

Cluster analysis resulted in three clusters that were statistically different from each other. These groups of farmers were characterised by differing attitudes towards need-based feeding. Sometimes attitude and management practice appeared to be consistent. For example, CL1 was the most uncertain in terms of nutrient requirements and was also the one with the lowest mean frequency of ration adjustments. CL2, on the other hand, considered regular ration adjustments as very important and had most feed analyses performed. With regard to another aspect, attitude (or cluster affiliation) did not have an effect, e.g., in terms of phase feeding. However, this may largely be due to the small sample sizes per animal species. The results show that organic farmers are far from being a homogenous group, but vary widely in terms of attitudes and beliefs, but also in terms of farm conditions and management practices.

Attitudes and beliefs are drivers of behaviour and need to be considered if behaviour change is intended. According to the COM-B model developed by Michie et al. [71], which has been used to better understand farmer behavior in regard to tail-biting in pigs [72], behaviour change depends on the capabilities, opportunities, and motivation of the respective individuals. In the case of need-based feeding, all of these aspects could be strengthened. Providing practical guidelines, determining efficiency indicators, and designing feeding tools specifically for organic animals and customisable to the individual farm or herd, coupled with advisory services, would address both the first two aspects (capabilities and opportunities). Introducing external economic incentives and establishing benchmarking systems based on the prevalence of diet-related production diseases, for example, would address the latter (motivation). A wide range of policy instruments exists to foster behavior change and improve animal welfare [73]. When developing and implementing management tools and advisory strategies, farmers’ attitudes and beliefs should be considered as well as their willingness to make the effort to collect and analyse data, plan feed rations, monitor results, etc. [74,75].

### 4.4. Limitations

As with any study, there are limitations to be reported. A total of 56 organic pig and poultry farms were visited during the project. Considering the framework of the project and the numerous activities conducted within, it was not feasible to include a larger number of farms. The sample was purposively selected to include a wide variety of species, feeding strategies, and attitudes. As recruitment was mainly carried out by advisors, it may have inadvertently included particularly committed and above-average farms, as well as farms in need of advice and therefore below-average. Therefore, the group may not be representative of organic pig and poultry farms in Germany.

The collection of data was partly dependent on self-reported information from farmers, which consequently leaves the data susceptible to subjective bias. Farmers may have overestimated or underestimated their own management practices and may have had a different understanding of certain issues. The same applies to the advisors involved in the data collection process. Despite thorough preparation and training, subjective bias may also have been present among this group.

The evaluation of the suitability of the rations was based on crude protein and energy levels. Indeed, within the project, amino acid profiles of single components were elaborately estimated using NIRS analyses and linear regressions established by Evonik Degussa [76], and amino acid profiles of total rations were calculated. Additionally, the in vitro ileal digestibility of the crude protein of the feed rations was determined using a multienzyme method [77]. However, because reference values were available mainly for crude nutrients, it was decided to use these for the evaluation.

PCA is generally regarded as a technique for large sample sizes. It has been shown, however, that sample sizes around or lower than 50 can be adequate when component solutions show high or mid-range component loadings with few components and large numbers of items [78] and communalities are high [79,80]. This was the case, which is why the sample size of 56 was accepted for PCA. Subsamples, particularly per species and type of production were, however, too small to perform further statistical analysis. Even where statistical analysis has been carried out, the results may not be generalisable. In this respect, it should be considered that diet-related dysfunctions and problems are highly farm-specific and therefore require context-specific solutions rather than general recommendations.

## 5. Conclusions

The aim of our study was to assess German organic pig and poultry farmers’ attitudes and beliefs towards ration planning to better understand drivers and barriers to the uptake of need-based feeding strategies. It was shown that 60% of the feed rations on the study farms deviated largely from recommendations, phase feeding was only partly implemented, and the majority of farms (85%) did not perform regular feed analyses. The survey revealed several uncertainties relating to the nutritional requirements of organic pig and poultry, the animals’ ability to compensate undersupply, and the effects of feeding on health and performance. Cluster analysis indicated three groups of farmers differing in their attitudes towards need-based feeding. Based on these results, our study identified the need for clarification of organic animals’ nutrient requirements as a means to eliminate farmers’ uncertainties in this regard. Available recommendations can be used but must be tailored to organic conditions and the animals kept in these systems. Low-threshold tools may be necessary that allow farmers to customise calculations and follow-up on changes, i.e., pinpoint the links between need-based feeding, performance, and health. Understanding farmers’ attitudes and beliefs may help advisors and other professionals in their efforts to support their clients. Overall, the understanding of farmer attitudes and beliefs can inform the debate on prospective approaches to improve need-based feeding in organic farming and, as a consequence, improve health and welfare in organic livestock systems.

## Figures and Tables

**Table 1:** Reference values for nutrient levels for different groups of animals (on an 88% DM basis).

Animals	Crude Protein (%)	Energy (MJ ME/kg)	Source ^1^
Sows from first litter			[18,20,24]
Sows gestating	12.0	11.5	
Sows lactating	18.0	13.5	
Piglets up to 30 kg			[18,20,24]
Prestarter (2–10 kg)	20.0	13.6	
Piglet feed I (10–20 kg)	18.0	13.0	
Piglet feed II (20–30 kg)	17.0	13.8	
Fattening pigs from 30 kg			[18,24]
Starter (30–60 kg)	18.5–20.0	13.0	
Grower (60–90 kg)	16.0	13.0	
Finisher (90–120 kg)	14.0	12.5	
Laying hens from start of lay			[19,25]
Layer I (up to peak lay)	18.0	10.9	
Layer II (from peak lay)	16.0	10.7	
Broilers			[21]
Starter	21.5	12.0	
Grower	20.0	11.3	
Finisher	18.0	11.0	
Turkeys			[20]
Starter (7–12 weeks)	26.4	12.0	
Grower (13–17 weeks)	-	-	
Finisher (18+ weeks)	18.0	12.0	

^1^ The exact reference values were not solely based on the sources listed but were complemented by the expertise of the farmers and their advisors.

**Table 2:** Sums of squared loadings, and variance explained for oblimin rotated four-component solution.

	Component 1	Component 2	Component 3	Component 4
Sums of squared loadings	3.29	2.54	2.58	2.30
Proportion variance	0.22	0.17	0.17	0.15
Cumulative variance	0.22	0.39	0.56	0.71
Proportion explained	0.31	0.24	0.24	0.22
Cumulative proportion	0.31	0.54	0.78	1.00
Component correlations				
Component 1	1	0.23	0.36	0.03
Component 2	0.23	1	0.27	−0.31
Component 3	0.36	0.27	1	0.03
Component 4	0.03	−0.31	0.03	1

**Table 3:** Component loadings, communality (h2), uniqueness (u2), and complexity (com) for oblimin rotated four-component solution for 15 Items (n = 56).

Item	Item (Statement)	Component Loading				h2	u2	com
		1	2	3	4			
14	Regular ration reviews and adjustments are economically imperative.	**0.90**	0.13	−0.03	−0.02	0.87	0.13	1.0
15	Individual components must be regularly analysed and rations recalculated.	**0.87**	0.06	−0.01	−0.01	0.78	0.22	1.0
11	Feed rations often have to be recalculated because the ingredients of the batches used change.	**0.84**	0.02	0.01	0.17	0.75	0.25	1.1
6	I am willing to spend additional time to optimize the feeding of my animals according to their needs.	**0.61**	−0.27	0.30	−0.08	0.54	0.46	1.9
13	Only the analyses of the single feedstuffs used allow for a good ration design.	**0.58**	−0.06	0.35	−0.20	0.60	0.40	1.9
2	Animals are able to compensate even with insufficient supply.	0.01	**0.79**	0.02	0.04	0.61	0.39	1.0
3	Good performance is possible even with a feed supply below the animals’ needs.	0.19	**0.75**	−0.07	0.00	0.63	0.37	1.2
5	Savings in feed purchases maintain the economic advantage, even when animals cannot deliver their maximum performance.	0.03	**0.74**	0.05	−0.06	0.62	0.38	1.0
16	Nutrient deficiencies do not harm the animals because they adapt their performance level to the feed offered.	−0.05	**0.69**	0.36	−0.15	0.80	0.20	1.6
4	The health of the animals is endangered by a supply that does not meet their needs.	0.03	0.12	**0.82**	−0.09	0.77	0.23	1.1
8	Animals of all ages must be fed according to their needs so that they perform at the best possible level.	0.09	−0.02	**0.82**	0.11	0.74	0.26	1.1
7	Providing animals of all ages according to their needs is important for their health.	0.05	0.12	**0.76**	0.15	0.70	0.30	1.1
10	The exact needs of the animals are difficult to determine.	0.08	0.07	−0.16	**0.89**	0.77	0.23	1.1
17	There are no uniform supply recommendations for organically reared animals.	−0.17	−0.05	0.24	**0.86**	0.82	0.18	1.2
9	The supply recommendations of the GfE ^1^ are guideline values that are not adapted to organic animal husbandry.	0.16	−0.19	0.05	**0.75**	0.72	0.28	1.2

^1^ GfE = German Society of Nutrition Physiology. Bold format marks the primary component loadings of all items.

**Table 4:** Farm characteristics of 56 organic pig and poultry farms.

		Sows	Fatteners	Layers	Broilers	Turkeys
	n	17	19	9	5	6
Herd size (places)	Mean	126	529	3736	1485	1437
	Min–Max	40–450	200–1300	375–15,000	200–4800	220–2700
Agricultural area (ha)	Total	71	297	165	191	65
	Arable	64	246	109	127	46
	Grassland	10	45	17	64	42
Feeding strategy	Self-mixing	4	17	5	-	1
	Purchase	5	2	4	1	2
	Both	6	-	-	1	3
	Unknown	2	-	-	3	-

**Table 5:** Performance data from 56 organic pig and poultry farms.

	Parameter	Median	Min	Max
Piglet producers (n = 17)	Herd size	76	40	450
	Piglets born alive	13.0	12.0	15.0
	Weaned piglets per litter	10.5	9.0	11.5
	Weaned piglets per sow and year	21.0	18.0	24.3
	Feed amount per sow and year (kg)	2280	1820	3680
	Preweaning death loss (%)	20.0	11.0	30.0
	Postweaning death loss (%)	2.0	0.5	5.0
	Replacement rate (%)	40.0	20.0	50.0
Pig fattening units (n = 19)	Herd size	485	200	1300
	Fattening period (days)	122	106	300
	Final fattening weight (live weight, kg)	126	117	150
	Daily weight gain (g)	790	433	850
	Feed conversion	3.1	2.8	4.8
	Feed use (kg/pig)	303	256	628
	Death loss (%)	1.8	0.5	8.0
Egg producers (n = 9)	Hens per laying period	4800	375	15,000
	Residence period (days)	425	365	567
	Hen-Housed Egg Production (eggs)	296	260	403
	Hen-Housed Egg Production (%)	76	66	90
	Egg mass (kg/hen housed)	19	17	26
	Feed use (kg/hen housed)	55	50	77
	Death loss (%)	8.0	1.0	15.0
Broiler farms (n = 5)	Broilers per fattening period	500	200	4800
	Fattening period (days)	84	65	112
	Final fattening weight (live weight, kg)	2.5	2.3	3.4
	Daily weight gain (g)	37.2	20.9	40.3
	Feed conversion	2.8	2.1	3.6
	Death loss (%)	3.0	1.5	5.0
Turkey farms (n = 6)	Turkeys per fattening period	1600	220	2700
	Fattening period of toms (days)	140	126	140
	Fattening period of hens (days)	104	98	140
	Final fattening weight mixed (live weight, kg)	12.3	10.6	13.9
	Feed use hens (kg/hen housed)	24	-	-
	Death loss (%)	4.0	2.0	15.0

**Table 6:** Crude protein and energy levels of feed components used by 56 organic pig and poultry farms (on an 88% DM basis).

Component	n	Crude Protein in %		Energy in MJ ME/kg	
		Mean	(Min–Max)	Mean	(Min–Max)
Faba beans	28	24.8	(22.2–27.4)	12.6	(12.2–13.1)
Peas	20	17.8	(12.5–23.1)	13.3	(12.5–14.2)
Barley	21	9.8	(7.7–11.9)	11.7	(11.5–11.9)
Oats	20	8.6	(5.7–11.5)	11.4	(11.2–11.7)
Triticale	19	9.3	(5.4–11.7)	11.9	(11.6–12.2)
Wheat	31	10.5	(8.8–15.1)	11.4	(9.0–13.5)

**Table 7:** Number of feeding phases on 56 organic pig and poultry farms.

Animals	n	No. of Farms with			Recommended No. of Feeding Phases	Source
		1	2	>2 Phases		
Sows from first litter	17	0	14	3	2+	[18,20,24]
Piglets up to 30 kg	17	6	7	4	3	[18,20,24]
Fattening pigs from 30 kg	19	4	8	7	2+	[18,24]
Laying hens from start of lay	9	8	1	0	2	[19,25]
Broilers	5	0	4	1	3	[21]
Turkeys	6	0	1	5	4	[23]

**Table 8:** Crude protein and energy levels of feed rations used by 56 organic pig and poultry farms (on an 88% DM basis).

Animals	Phase	n	Crude Protein in %		Energy in MJ ME/kg	
			Mean	(Min–Max)	Mean	(Min–Max)
Sows	Gestating	17	13.2	(8.8–15.9)	12.1	(10.4–13.4)
	Lactating	17	16.7	(15.1–20)	12.7	(11.4–13.6)
Fattening pigs	Starter	19	17.1	(15.2–20.5)	13.3	(12.2–14.9)
	Grower	14	14.7	(12.4–18.3)	13.1	(12.1–14.6)
Laying hens	Universal	9	16.7	(15.4–18.8)	10.5	(8.9–12.5)
Broilers	Universal	5	18.0	(14.9–20.8)	11.5	(9.3–12.7)
Turkeys	Starter	6	22.6	(18.5–28)	11.6	(11.2–12.2)
	Finisher	6	16.6	(13.7–18.7)	11.7	(11.1–12.6)

**Table 9:** Cluster-forming variables of the three clusters.

Cluster-Forming Variables ^1^	Cluster 1	Cluster 2	Cluster 3	*p*-Value
	n = 28	n = 11	n = 17	
Component 1—Regular adjustment	0.54 ± 0.64 ^a^	1.42 ± 0.39 ^b^	0.12 ± 0.60 ^a^	<0.001
Component 2—Compensation (reverse)	0.01 ± 0.65 ^a^	1.16 ± 0.57 ^b^	0.69 ± 0.36 ^b^	<0.001
Component 3—Health and Performance	0.80 ± 0.70 ^a^	1.94 ± 0.13 ^b^	0.55 ± 0.41 ^a^	<0.001
Component 4—Uncertainty	1.12 ± 0.54 ^a^	0.39 ± 0.83 ^b^	−0.18 ± 0.41 ^c^	<0.001

^1^ Mean values ± standard deviation of composite scores (ranging from −2 to 2), values sharing a letter in the group label are not significantly different at the 5% level.

**Table 10:** Distribution of animals among the three clusters.

Animals	Cluster 1	Cluster 2	Cluster 3
	n = 28	n = 11	n = 17
Fattening pigs	4	4	9
Piglet production/sows	9	2	6
Pigs (closed system)	2	0	0
Laying hens	7	1	1
Broilers	3	1	1
Turkeys	3	3	0

**Table 11:** Cluster-describing variables of the three clusters.

Cluster-Describing Variables	Cluster 1	Cluster 2	Cluster 3	*p*-Value
	n = 28	n = 11	n = 17	
Agricultural area (ha) ^1^	134 ± 180	67 ± 56	107 ± 155	0.313
Time since conversion (years)	14 ± 11	9 ± 5	14 ± 10	0.272
Division of responsibilities crop/animals (% of farms)	29	36	24	0.764
Importance of farm branch ^2^	3.7 ± 1.4	3.9 ± 1.5	3.7 ± 1.3	0.903
Feed management				
Verification of feed declaration ^3^	1.3 ± 0.4	1.5 ± 0.7	1.1 ± 0.2	0.119
Frequency of feed analyses ^4^	2.1 ± 1.0 ^a^	3.1 ± 1.3 ^b^	2.6 ± 1.1 ^ab^	0.067
Frequency of ration calculation ^4^	2.2 ± 1.0 ^a^	3.1 ± 1.0 ^b^	2.9 ± 1.2 ^b^	0.010
Consideration of ileal digestibility of amino acids ^4^	1.0 ± 0.0 ^a^	2.6 ± 1.9 ^b^	2.1 ± 1.3 ^b^	<0.001
Phase-feeding				
Mean number of phases in fattening pigs ^5^	2.0 ± 0.9	3.0 ± 0.8	2.1 ± 0.9	0.244
Mean number of phases in laying hens ^6^	1.1 ± 0.4	1.0	1.0	-
Performance measures				
Weaned piglets per sow and year ^7^	19.6 ± 6.0	20.6 ± 0.9	20.9 ± 2.1	0.854
Daily weight gain fattening pigs (g/d) ^5^	752 ± 53	821 ± 48	799 ± 71	0.273
Eggs per hen and year ^6^	269 ± 10	292	-	0.132
Mentioning of problems (% of farms)				
Feather pecking, tail biting, cannibalism	24.0	10.0	12.5	0.450
Feacal consistency, diarrhoea	12.0	0.0	18.8	0.352
Performance	16.0	0.0	0.0	0.105

^1^ one outlier removed (3.500 ha), ^2^ scaling: 1 = low to 5 = high, ^3^ scaling: 1 = none/complete trust, 2 = random sampling, 3 = complete sampling, ^4^ scaling: 1 = none/no to 5 = all feeds annually/ration calculation several times a year/yes, based on analyses for each feed component, ^5^ only pig-fattening farms (n = 19), ^6^ only egg-producing farms (n = 9), ^7^ only piglet-producing farms (n = 17), values sharing a letter in the group label are not significantly different at the 5% level.

## Data Availability

Data are contained within the article. Raw data are available from the corresponding author upon reasonable request.

## Abbreviations

The following abbreviations are used in this manuscript:

CL	Cluster
GfE	German Society for Nutritional Physiology
KMO	Kaiser-Meyer-Olkin criterion
PCA	Principal component analysis
